# Controversial views and moral realism

**DOI:** 10.1007/s11017-023-09616-4

**Published:** 2023-03-03

**Authors:** Henrik Rydenfelt

**Affiliations:** University of Helsinki, Helsinki, Finland

**Keywords:** Controversy, Moral realism, Pragmatism, Expressivism, Anti-realism, Fallibilism, Humor

## Abstract

It is argued that the emergence of controversial views in discussions of theoretical medicine and bioethics is best explained by the assumption of moral realism within those discursive practices. Neither of the main alternatives of realism in contemporary meta-ethics — moral expressivism and anti-realism — can account for the rise of controversies in the bioethical debate. This argument draws from the contemporary expressivist or anti-representationalist pragmatism as advanced by Richard Rorty and Huw Price, as well as the pragmatist scientific realism and fallibilism of the founder of pragmatism, Charles S. Peirce. In accordance with the fallibilist view, it is proposed that presenting controversial positions may serve epistemic purposes within bioethical debates, providing opportunities for inquiry by pointing towards problems to be solved and arguments and evidence for and against to be put forward.

## Introduction

Bioethics and medical ethics are often referred to as examples of ethical debates and discussions that involve long-term, persistent disagreements and stark controversies between opposing camps. Issues of life and death, euthanasia, abortion, donation of organs, transplants, artificial life, and so on, sometimes result in views that spark lively debates, conflicts of opinion, and even public outrage. This state of affairs is hardly remarkable, and in bioethical debates, the emergence of controversial views is often taken as a matter of course. However, the fact that these discussions are susceptible to producing such positions is not inconsequential for thinking about the nature of bioethical discourse. Philosophers have long debated whether discussions in normative and applied ethics entail or preclude commitments to one or another meta-ethical stance, and what implications – if any – such commitments have concerning the viability of meta-ethical views. What sort of implications does the emergence of controversial views have concerning the epistemological and ontological assumptions involved?

In this article, I will argue that the existence of controversial views in discussions of theoretical medicine and bioethics is best explained by the assumption of moral realism within those discursive practices. This is not an argument to the effect that moral realism is the case. In my view, it is doubtful that there could be a way to provide a “proof” of realism that is neither circular nor guilty of an infinite regress; the same is the case with the opposing theses of moral anti-realism or nihilism, or forms of moral skepticism that maintain that one cannot have moral knowledge. Instead, I will limit my considerations to defend the more modest conclusion that (at least some) ethical discussions and debates involve the assumption of realism, as evidenced by the emergence of controversial views. After presenting this argument, I will consider some of its implications for bioethical debates and argue that the presentation of controversial views may serve various epistemic purposes within such discursive practices. To make this argument, I will draw from the tradition of philosophical pragmatism. Since its inception in the late 1800s by the philosopher and polymath Charles S. Peirce, pragmatism has come in many stripes and forms. The two stripes that will be of interest here are the contemporary expressivist or anti-representationalist pragmatism as advanced by Richard Rorty and Huw Price, as well as Peirce’s own pragmatist scientific realism. As my intent is to make a general point concerning a feature of the discursive practice of bioethics, I will not focus this discussion on any particular controversial view(s); instead, I will consider the broader phenomenon of the emergence of such views in terms of a definition presently provided.

### The notion of a controversial view

The literature on bioethics — as well as public discussion on its themes — includes morally controversial issues (i.e., topics where there appears to be widespread disagreement on moral grounds). Controversial *views*, as I intend to use the term here, are distinct from controversial issues. Such views typically spark disagreement by going against the views of the majority and are typically voiced concerning issues that are not yet controversial – when there is a common or widely shared position that the controversial view aspires to contest.^1^ Consider a strong antinatalist proposal that having children is immoral [1], or an argument that supports gestational donation with embryos transferred to brain dead donors [2]. Such views go counter common prevailing views and practices and have sparked vehement disagreement on part of others. But they also call for the articulation of cogent responses that often result in complicated debates. Why should one oppose gestational donation, if it does not introduce issues other than those involved in other forms of organ donation that receive wide acceptance? If one is (at least to an extent) motivated by utilitarian considerations, why would one produce children who will inevitably face suffering that would otherwise be avoided? Responses to these questions require digging into the intricacies of one's background assumptions, sometimes revealing some of their apparent mistakes.

For the purposes of this discussion, let me propose a definition of a controversial view in light of the considerations just presented – one that appears to fit well with the way the notion of a controversial view is used in scientific and philosophical debates. This definition includes two necessary and jointly sufficient conditions which controversial views are expected to satisfy. The first is that the view in question is one over which there is genuine disagreement. The second condition is that the view in question is not easily dismissed with reference to commonly accepted starting points – conceptualizations, theoretical starting points, standards and norms, and the like–of the discussion or debate in question.

Both conditions deserve further elucidation. The notion of a genuine disagreement that the first condition involves is particularly salient. What is meant by genuine disagreement, in this context, is the existence of a difference of opinion that is taken to call for a resolution. A genuine disagreement differs from a disagreement that does not appear to require resolution at all, such as a “disagreement” over a matter of taste. Concerning matters of individual taste and preference, disagreements do not matter; although, in some cases, the contrary views of others may be viewed as a result of deviance in individual preferences best handled by therapeutic means, as one might describe a taste for raw bell peppers. In a genuine disagreement, by contrast, there is usually the felt need to voice and argue for a contrary opinion, even if that opinion appears to be widely shared. While it may sometimes be tempting to criticize controversial proposals on the grounds that the authors are merely deviant or even insane, the same proposals are deemed deserving of careful counter-argumentation (by those among the relevant discussants or “experts”). The raging debate on antinatalism may serve as a case in point.

The second condition may be considered too limiting. Many views are superficially “controversial” in that there is active and entrenched disagreement concerning them in public discussion. What is sometimes called a controversy may arise from the presentation of a position that is patently stupid or violates commonly accepted norms of justification. However, such controversies do not involve a controversial view as presently intended. What makes, or keeps, a view controversial is that it is not easily dismissed (at least at present) by reference to commonly accepted conceptualizations, theoretical starting points, and norms of justification. Instead, such considerations – at least as they are – appear insufficient to immediately show that the presented view is mistaken or erroneous. If one is not prepared to accept gestational donation on the same grounds that one approves of the donation of other organs, one owes an explanation; –that is, an account of why the case is different, or of how one's earlier view of the justification of organ donation was mistaken or incomplete.

Notably, both conditions implicitly refer to a community; without the existence of such a community, no genuine disagreement could take place, nor would there be any commonly accepted starting points. In a case of scientific controversy, the relevant community is the scientific community which considers the relevant issues. The borders of the relevant community are often not clear-cut, and the debate concerning a controversial view may well expand those borders, as well as invite questions over their precise location. Nevertheless, one clearly engages in discursive practices where the assessment of a particular community is relevant.

Finally, the definition does not exclude the presentation of a controversial view that one does not oneself fully believe or subscribe to. The disagreement in question may be more “academic” in nature, and the aim of putting forth a controversial view may be to make an issue controversial. In particular, the presentation of a controversial view may be ironic (as in Jonathan Swift’s “Modest Proposal” to sell the children of poor Irish families to be eaten by the rich discussed in Matti Häyry’s article in this issue [3]). The controversial position may be put forward in order to advance an argument *ad absurdum* concerning some commonly accepted starting points (as in Häyry’s exposition of Swift’s argument). Even when ironically presented, a controversial view may invite genuine disagreement, while it is not easily dismissed by drawing from shared starting points. With these considerations in place, I will now examine the implications of the existence of controversial views in moral ontology and epistemology.

### Disagreement and moral facts

The fact of persistent disagreement has often been evoked in arguments against realism. It has been proposed that persistent disagreement and the change of expert and public opinion on certain topics suggests that there are no facts of the matter at stake concerning the issues in question. In particular, disagreement over moral views over the centuries has been suggested to imply that there are no moral “facts” [4]. At a minimum, moral realists maintain that there are moral facts, and that moral claims or judgments are true if they get the moral facts right; hence entrenched disagreement shows (it has been claimed) that moral realism is mistaken. As sharp cases of disagreement, the emergence of controversial views could be argued to imply that any position that currently receives wide support from the experts and the public can eventually be turned into a point of debate, counting against a realist view. However, the fact of disagreement may also be evoked in an argument to the effect that realism is a prevailing position about an issue at hand. Does not disagreement over issues in bioethics and medical ethics, for example, suggest that one presupposes that there is some fact of the matter at stake in these debates? Does not controversy imply that one have accepted (at least some form of) moral realism?

The alternatives of realism in contemporary meta-ethics — moral anti-realism and expressivism – have different views of whether such realist assumptions require an explanation. Moral anti-realists maintain that there are no moral facts, and that one's moral judgments and assertions (purporting to represent such facts) are severally mistaken [4]. The emergence and existence of controversial views appear to show that moral anti-realism is the prevailing view within current discursive practice. Perhaps one could somehow maintain moral views despite thinking that they are all in error. But why would one disagree with the views of others if none is the wiser? I think this is a potent argument against moral anti-realism as something that one is committed to in practice. However, it is not a proof of its untruth. Indeed, many anti-realists have argued that most people are not aware of the erroneousness of their moral claims, and, rather, subscribe to a realist view [4, 5]. Typically, the anti-realists do not even contest the view that one's discursive practices involve realist assumptions.

Moral expressivists maintain that moral judgments or assertions are not, despite appearances to the contrary, in the business of reporting or representing facts. Rather, moral judgments are expressions of feelings, desires and other conative states or stances which do not attempt to reflect the world as it is [6]. For this reason, moral judgments are not mistaken; they simply do not describe the world. Expressivists have sometimes been counted in the “anti-realist” camp of meta-ethics. However, as contemporary expressivists may not make any direct claims *denying* the existence of moral realities or “facts,” I will here use the label of anti-realism to refer to views already discussed. Unlike anti-realism, expressivism remains a genuine contender. Expressivists have attempted to explain the various features of discursive practices without reference to either moral facts or to assumptions, within those practices, concerning such facts [6]. For these purposes, they have often resorted to semantic deflationism or minimalism, which eschews the notion of truth or reference as a relation between, say, words and realities, and concentrates on the function of the truth predicate as a linguistic device.

However, even equipped with a deflationary account of truth, the expressivists face a problem in explaining moral disagreement. Semantic deflationism helps to explain how words such as “is true” (or perhaps, “is a fact”) can be attached to moral claims that are expressions of desires, preferences, and the like. But if one *thought* that moral claims and judgments are such expressions, why would one not treat them analogously to matters of taste not worthy of genuine disagreement? Expressivists and their precursors such as meta-ethical emotivists have for a long time attempted to show why disagreement over attitudes would be be treated analogously to disagreement over fact [6, 7]. However, a succinct and probably the most promising response to this problem has been developed in discussions of contemporary anti-representationalist pragmatism. Anti-representationalism can be viewed as the extension of expressivism to a global scale: the anti-representationalists maintain that the task of thought and talk in general is not to represent reality or reflect the way the world is. Nevertheless, Huw Price has argued that, in many discursive practices, a norm of truth is present aside the norms of honesty and justification [8]. Calling someone’s statement true is not merely to reassert or endorse that statement, but also to say that the speaker is correct. This assessment of correctness takes place with reference to whether one is prepared to make the same or a contrary assertion: “We are prepared to make the judgement that a speaker is incorrect, or mistaken, in this sense, simply on the basis that we are prepared to make a contrary assertion; […]” [8, p. 176]. Imagine, for contrast, a linguistic community where this norm of truth is absent. For the speakers in this community, disagreement does not matter. If the norm of truth is not in place, “the wheels of argument do not engage; disagreements slide past one another” [8, p. 185–186].

Price’s discussion offers an convenient way for the expressivist to deal with the issue of disagreement without reference to realist assumptions. In order to spark disagreement, it is only required that the norm of truth is present in one's discursive practice (concerning the issue at hand). However, no assumptions concerning realism need to be invoked: it suffices that one's discursive practices involve a simple norm of truth. This norm, in turn, may have developed simply due to the practical need to coordinate one's various stances (such as moral views). In this way, the genuine disagreement that controversial views involve can be explained without reference to realist assumptions. It is the second condition of controversial views that expressivists will find more difficult to explain, as I will now turn to argue.

### Fallibilism and the realist assumption

According to the second condition of a controversial view, such views are not easily dismissed as an error or a mistake in light of commonly accepted starting points. In this section, I will consider how a pragmatist version of moral realism can explain this feature of discursive practices, and then argue that the emergence of controversial views that satisfy this condition is not similarly explainable by expressivist means. In the next section, I will consider the prospects of alternative views in moral epistemology in providing a feasible explanation.

A second prominent line of pragmatist thinking is its version of scientific realism. Not all pragmatists are realists — indeed, many notable pragmatists such as Joseph Margolis [9] have defended sophisticated forms of relativism, while anti-representationalist pragmatists have deliberately attempted to circumvent the whole issue of realism and anti-realism. However, from its very inception, pragmatism has also been linked with a sophisticated form of scientific realism advanced by Charles S. Peirce [10]. In Peirce’s view, in some practices of settling and justifying opinions, one aims to ascertain how things truly are. These practices he referred to as science and distinguished them from all others by the fundamental hypothesis that there are real things “whose characters are entirely independent of our opinions about them” [10, p. 120].^2^ This realism — which I have elsewhere called *hypothetical — *does not profess to prove that there are real things, or that science is the best guide to reality [11, 12]. Instead, it merely points out that in some discursive practices, one is committed to this realist assumption. As truth is independent of anyone’s opinion, any opinion may be mistaken. Moreover, the presence of this realist assumption in a discursive practice is indicated by *fallibilism*: even one's best justified opinions are not considered the final word on the issue.

Take a view that is controversial but carefully presented – say, a strong antinatalist proposal or an argument that supports gestational donation with embryos transferred to brain dead donors. As already pointed out, these controversial views may be presented for the sake of the argument, or in an ironic vein. However, even then they are not easily dismissed as simple mistakes. The fact that the contrary position appears justified and is shared by the majority within the relevant community – sometimes even by those who present the controversial view – is not sufficient to settle the issue. This is a first trace of fallibilism; the common, justified opinion of the majority – in many cases including the relevant experts – is not decisive. A second trace of fallibilism is evidenced by the fact that controversial views are prone to raise doubt concerning the commonly accepted starting points. In some cases, despite going against shared conceptualisations and/or norms and standards, a controversial view appears to be justified and difficult to dismiss. If the commonly accepted starting points were beyond doubt, such a situation should not arise, and the controversial view should be easily deemed mistaken. Other controversial views extend the application of such starting points to a new case, arriving at a conclusion that appears unacceptable. In such cases, if one did not treat the subject in a fallibilist manner, the controversial view should appear as an acceptable consequence of one's starting points. The pragmatist realist has a simple explanation to offer: it is because one assumes that the fact of the matter concerning, say, whether it would be right to enable gestational donation, is independent of the commonly accepted views and starting points of that discourse, that a controversial view concerning that issue may emerge.

This second feature of controversial views, that they cannot be simply rejected as mistakes in light of commonly accepted starting points, is something that the expressivist will find difficult to explain. Arguably, it is something that expressivists have not even attempted to account for. Expressivism does not deny that everyone is a fallibilist – or that one should resort to, say, expert consensus in settling one's opinions over matters of ethics – expressivism typically aspires to be neutral when it comes to such issues of justifying and settling moral views. Without suitable provisos in place, however, simply admitting that the realist assumption is in play in (at least some) moral debates would fly in the face of the very expressivist *credo* that one's moral claims are expressions of conative states or stances that do not aspire to reflect or represent realities.^3^ To produce such a proviso, the expressivist could argue that the realist assumption is an error introduced at some point to one's discursive practices – an overestimation of what one is doing (or can be doing) when engaged in one's moral debates. The resulting view would not be equal to the anti-realist position already discussed; as one's moral claims remain expressions of conative states or stances, they are not severally mistaken. Rather, the error resides in what one thinks one's moral discourse may achieve. Importantly for the present argument, however, this adaptation of expressivism – just like anti-realism–does not deny the presence of the realist assumption (erroneous as it might be).

### Epistemological alternatives

In the previous section it was argued that the emergence of controversial views in theoretical medicine and bioethics can be explained by an assumption of moral realism in the discourse. If moral expressivists draw from this assumption to explain the features of one's discursive practices, their position needs considerable modifications. However, an explanation for the emergence of controversial views might be sought from other quarters. Expressivists might attempt to provide a competing explanation; one that does not involve the realist assumption. Similarly, some of those who (unlike expressivists) think that moral claims express beliefs could argue that the emergence of controversial views can be explained without recourse to the assumption that one's moral claims aim to reflect realities that are independent of anyone’s opinions.

Consider the venerable tradition that maintains that moral truths are not made true by moral facts but are *a priori *— for example, Immanuel Kant’s view that the categorical imperative is binding solely as the dictate of one's rational will [13].^4^ These views maintain that moral truths are not dependent on what one may — actually and currently — maintain, while they also contest the idea of “moral facts,” and do not maintain that moral truths reflect mind-independent realities. Even *a priori *truths need not always be self-evident in the sense that one could immediately ascertain their truth by simply considering the proposition in question. Many of the same philosophers maintain that mathematical truths are *a priori*; however, one may easily be mistaken in one's calculations, and in many cases, one cannot immediately tell the solution to a mathematical puzzle (say, a complex equation). Proponents of *a priori* accounts of morality could attempt to explain controversial views as mistakes that arise from one's limitations in discovering *a priori* truths. Perhaps one's troubles in dismissing some controversial views as mistakes are due to the fact that one cannot tell, at the outset, whether the controversial view is an *a* *priori* truth (or a logical consequence of *a priori* truths), or whether the reasoning (or rational intuition that produced that view) is simply mistaken.

However, explaining the emergence of controversial views as a consequence of either one's ignorance or erroneous reasoning seems tenuous at best.^5^ Controversial views do not appear to be due to lapses of judgment, nor are they dismissable as errors analogous to miscalculation. Rather, they have the potential to challenge commonly accepted starting points that otherwise could be taken to enjoy the status of *a priori* truths. To account for this fact, proponents of *a priori* moral truths could refer to a related family of views in moral epistemology, sometimes called (moral) constructivism, that provides a more complicated view of the way in which moral truth (sometimes referred to as the “validity” of moral views) is dependent on one's opinions. Constructivists maintain that the true, correct or valid moral views are those that one would arrive at under some normal or ideal conditions. Proposals for such conditions include, for example, rationality, reflective equilibrium [14], reasoned reflection cleansed of the influence of individual interests and preferences [15], or acceptability without coercion in a sufficiently reasonable discourse [16]. The fact that controversial views are difficult to dismiss, they could argue, merely indicates that one's theoretical views and standards may be different from those that one would have under some more ideal conditions – when no more controversial views would appear. In this fashion, the constructivist can attempt to explain the emergence of controversial views by the distance between current and ideal conditions.

This explanation, however, faces considerable difficulties. Consider controversial views that extend shared starting points to new cases and arrive at conclusions that are seemingly unacceptable. Such views do not contradict one's current, reasonable-seeming starting points, nor do they point to an inconsistency within them. Nevertheless, they may raise doubts about those starting points. For example, the principles commonly accepted in organ donation appear to cover gestational donation. However, the application of these principles to the novel case may result in sufficient controversy to cause concerns about the soundness of one's initial, commonly accepted principles. Faced with such a case, the constructivist may retort that this is only to be expected; ideal conditions are difficult to achieve, and perhaps cannot be spelled out in advance with any amount of philosophical refinement. For this reason, one may never conclude that one might achieve a state in which no controversial views would arise. But this response appears to be equivalent to admitting that there are no conditions, no matter how ideal, where one's reasonable-seeming views are not similarly vulnerable — that any coherent and rational set of shared views arrived at by reasoned reflection and discourse can similarly be brought under doubt. This is, in effect, to admit fallibilism, the core of pragmatist realism.

The alternatives here briefly considered are obviously a small selection of possible stances in moral epistemology, and even in the family of *a priori* views. However, they also seem to be the main alternatives to the assumption of moral realism. If accounts of moral truth as *a priori* or as the product of reasoning under some normal and ideal conditions cannot account for the emergence and nature of moral controversies, it remains to be seen what shape an alternative view that could provide such an explanation might take.

## Conclusion

If the argument of the previous sections is successful, the existence of controversial views in discursive practices is best explained by the presence of an assumption of moral realism. While the fact of disagreement may be explained by the moral expressivist by drawing from Price’s norm of truth, the fact that controversial views are not easily dismissed as mistakes appears difficult to explain by alternative approaches to moral epistemology and ontology without drawing from the realist assumption. Assume, then, that moral realism is a prevalent assumption in those discursive practices that involve controversial views. In practice, what follows?

One consequence is that, in practical contexts, the emergence of controversial views can be treated as an indicator concerning the epistemological and ontological commitments of the discursive practice at hand. A key lesson of the pragmatist approach is that it is an empirical question – one that does not call for an *a priori* answer arrived at by armchair reflection – whether one is a fallibilist, and maintains the realist assumption, over any particular issue within a particular discursive practice. Taken as a whole, discursive practices may include topics that do not involve the realist assumption. Discussions on medical ethics sometimes broach topics that are currently considered to be matters of taste or preference and concerning which disagreements do not arise. There are doubtless also morally laden issues that are settled, in one's present practices of bioethical discourse, by relying on the common starting points and expert consensus. Neither kind of topic is, at least for the time being, prone to spark controversial views in the sense intended here. (not incidentally, one is unlikely to call these topics *moral* or *ethical* issues or concerns, at least at present). However, concerning other issues, controversial views arise, indicating that these issues are treated in the realist fashion identified here. In this way, controversial views may reveal boundaries and patterns within discursive practices.

Another consequence is that the presenting of controversial views may incur changes in the contours of one's practice. An issue might first appear as a matter of taste, but upon the presentation of a controversial view, it may turn out to involve the realist assumption after all. Imagine that one maintained, quite innocuously, that deciding on what happens to one’s body after death is a question of personal and cultural preference. It is good to be buried in a box underground, some say, while others maintain that the decent thing is to be turned into ashes; no controversy arises. However, imagine that someone wishes one’s carcass to be placed in their home window, in full view of everyone on the street. A controversy may arise (what of the children passing by?). In this way controversial views may reveal, or perhaps even introduce, the realist assumption in one's practice.^6^ Finally, a third consequence flows from the capacity that controversial views hold for accepted starting points. Presenting such views may be beneficial in providing opportunities for inquiry by pointing towards problems to be solved and arguments and evidence for and against to be presented.

These consequences suggest that the presentation of controversial views may in many cases have considerable epistemic benefits, even when such views are expressed without subscribing to them, for solely academic purposes or in an ironic fashion. They recommend a form of tolerance towards such views, motivated by epistemic purposes.^7^ Such tolerance, however, need not extend similarly to every instance. Controversial views are also produced and presented in light of interests other than those of advancing one's (moral) inquiries. For example, controversy may be stirred in order to garner attention and gain publicity, or to advance parochial views without a commitment to inquiry, or simply for the sake of controversy. Perhaps, in some cases, such actions are justifiable on other grounds, but they cannot be justified with reference to the attempt to deploy the epistemic powers of controversial views here distinguished.

## Footnotes

1 Both notions are to be kept distinct from the notion of moral dilemma. The existence — if any — of moral dilemmas has considerable implications concerning moral epistemology. However, these implications are not among the ones considered here. Moral dilemmas are not the same as moral controversies; indeed, it might be wholly uncontroversial that a moral dilemma exists.

2 Elsewhere I have argued that this scientific realism is consistent with the global expressivist position of Rorty and Price; see [11].

3 The pragmatist realist may grant and, indeed, draw from the central expressivist and anti-representationalist point that one's claims or judgments do not represent realities simply in virtue of being claims or judgments. For discussion on this point, and on how pragmatist realism can remain anti-representationalist, see [11].

4 It might be tempting, for the proponent of an *a priori* account of moral truths, to attempt to explain controversial views as being due to individual preferences and feelings that cloud the judgment of those presenting such views. However, controversial views are controversial in that they are not taken to be solely the products of such individual factors; rather, they are treated as points of genuine disagreement.

5 Simplistic moral intuitionism, according to which the moral features of actions and situations are detectable by a specific type of “intuition,” is similarly vulnerable. If such a view of moral epistemology was the one maintained in practice, controversial views would be extremely unlikely to emerge.

6 It may even be that controversial views, in some cases, not only show that the realist assumption is in place, but turn an issue into a realist on. However, further consideration of this possibility is beyond my scope here.

7 Such tolerance is connected with another tenet that pragmatism has long been associated with – pluralism. As a normative thesis, pluralism maintains, roughly, that differences in opinion are something to be tolerated, at least to an extent. Pluralism is not intended to imply that ethical issues are hopelessly intractable or plural in the sense that it depends on one’s point of view; it is not the same as relativism [17]. Instead, many pragmatists have held — although in different ways — that ethical questions and problems are amenable to inquiry that is (or at least closely resembles) science [11, 12, 18–20]. From the point of view of these pragmatists, diversity of opinion, when tolerated — in some cases, perhaps even encouraged — may help to advance one's inquiries [21, 22]).
